# Supplementary material for: The molecular principles underlying diverse functions of the SLC26 family of proteins
Source: J Biol Chem. 2024 Apr 4;300(5):107261. doi: 10.1016/j.jbc.2024.107261 (PMC11078650; doi:10.1016/j.jbc.2024.107261)
Supplement: Supporting Figures S1–S3 [file mmc1.pdf]

SUPPORTING INFORMATION

The molecular principles underlying diverse functions of the SLC26 family of proteins

Satoe Takahashi and Kazuaki Homma

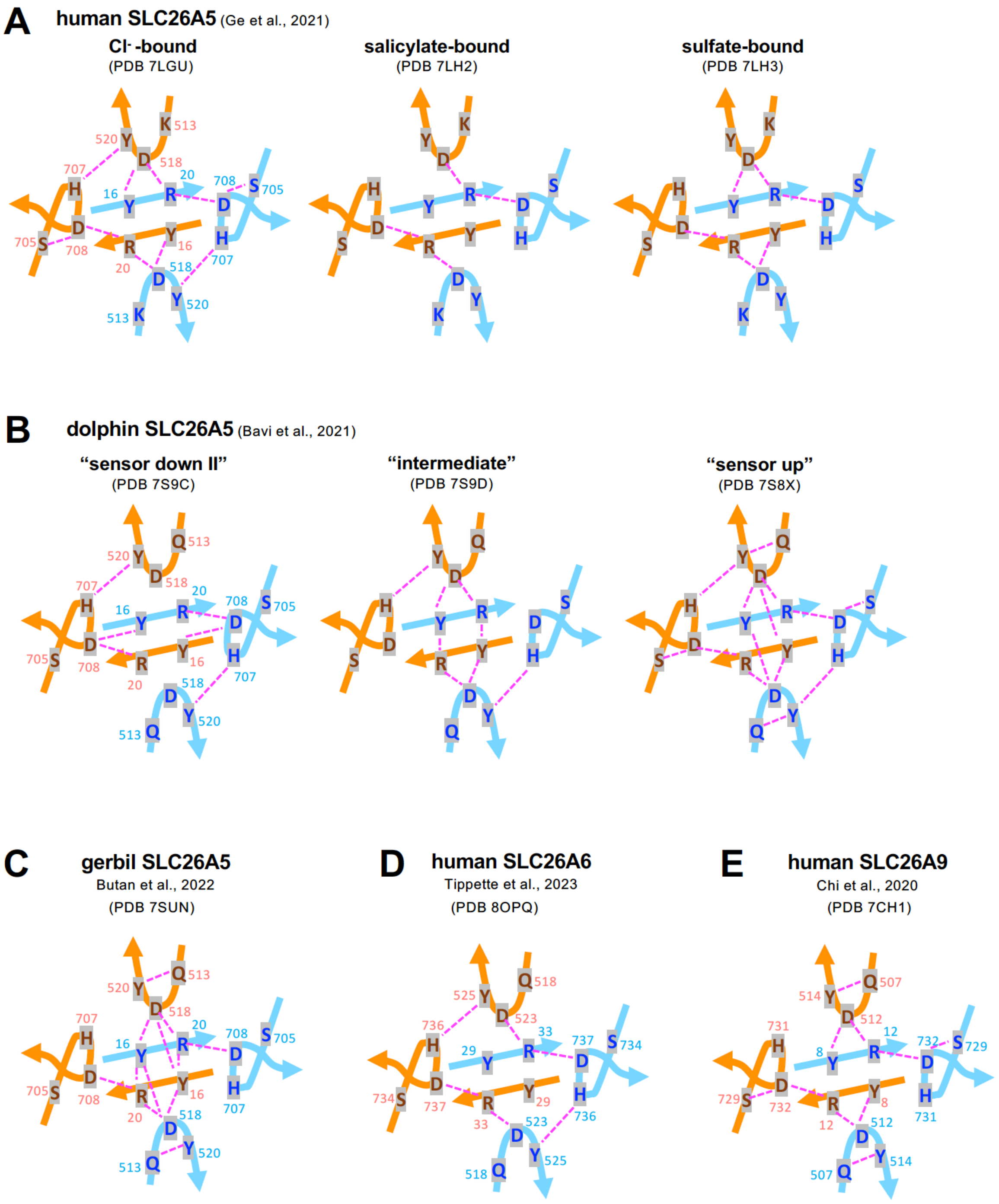

Figure S1. Schematic representations of the N- and C-terminal interactions in human SLC26A5 (A), dolphin SLC26A5 (B), gerbil SLC26A5 (C), human SLC26A6 (D), human SLC26A9 (E). Magenta dashed lines indicate potential hydrogen bond interactions. This supplementary figure is related to Fig. 1 in the main text.

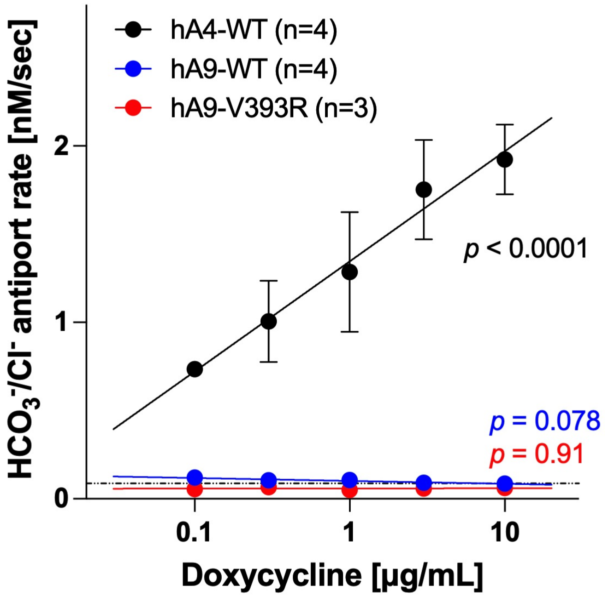

Figure S2. The V393R missense change does not confer HCO_3^-/Cl^- antiport activity on SLC26A9. HCO_3^-/Cl^- antiport rates were measured as in Fig. 6 using HEK293T cells expressing hA4-WT (positive control), hA9-WT, or hA9-V393R in a doxycycline (Dox)-dependent manner and plotted against Dox concentrations used. A horizontal dashed line indicates the basal transport rate of non-induced cells (negative control). Error bars indicate standard deviation. Solid lines indicate linear regressions (log_{10}[Dox] vs. transport rates). F-tests found a Dox-dependent increase in the anion transport activity for hA4-WT (p <0.0001) but not for hA9-WT (p = 0.078) or hA9-V393R (p = 0.91), indicating that V393R does not confer HCO_3^-/Cl^- antiport activity on SLC26A9.

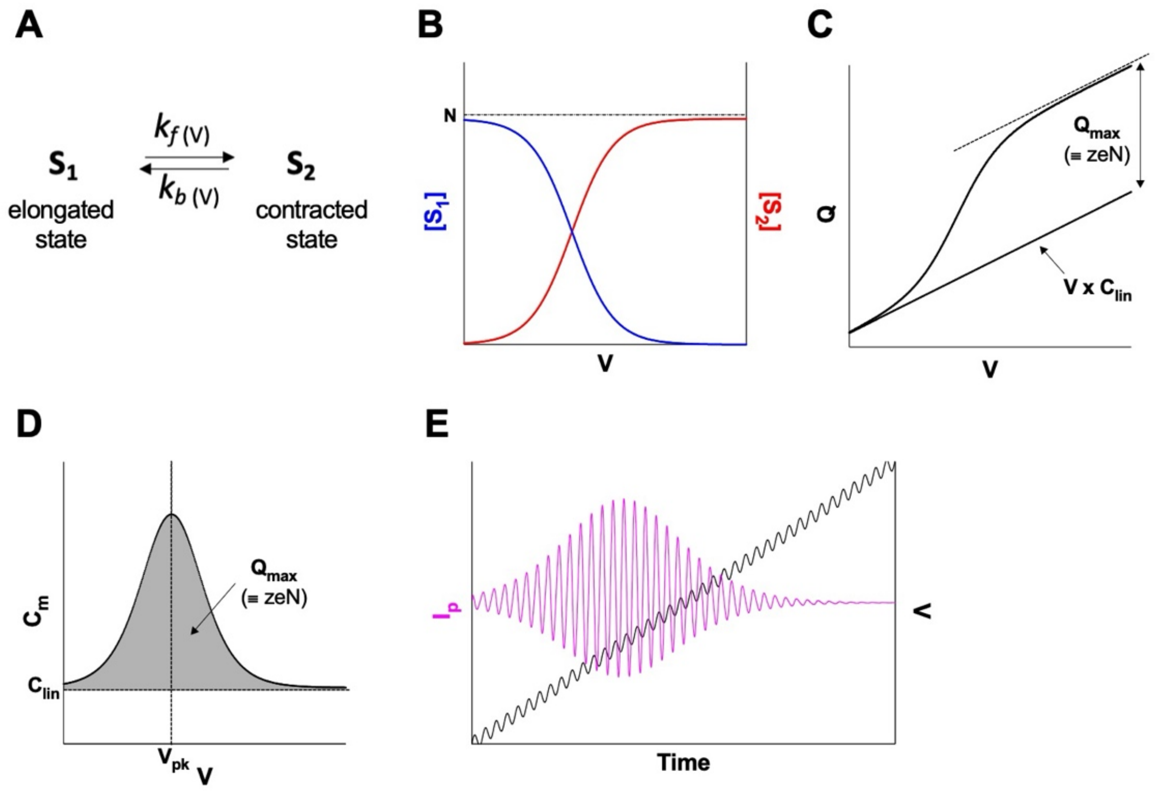

Figure S3. Nonlinear capacitance of prestin. (**A**) S_1 and S_2 represent voltage-dependent conformational states of prestin. Transitions between these two states are governed by the two voltage-dependent rate constants, $k_{f(V)}$ (Eq. 4) and $k_{b(V)}$ (Eq. 5). (**B**) A graphical representation of Eqs. 9 and 10. (**C**) A graphical representation of Eq. 11. (**D**) A graphical representation of Eq. 1. (**E**) A prestin-associated current, I_p (magenta), elicited by a ramped sinusoidal voltage stimulus (black).
